# Prevalence of antifolate resistance mutations in *Plasmodium falciparum* isolates in Afghanistan

**DOI:** 10.1186/1475-2875-12-96

**Published:** 2013-03-15

**Authors:** Ghulam R Awab, Sasithon Pukrittayakamee, Natsuda Jamornthanyawat, Fazel Yamin, Arjen M Dondorp, Nicholas PJ Day, Nicholas J White, Charles J Woodrow, Mallika Imwong

**Affiliations:** 1 Mahidol-Oxford Tropical Medicine Research Unit (MORU), Faculty of Tropical Medicine, Bangkok, Thailand; 2 Department of Clinical Tropical Medicine, Faculty of Tropical Medicine, Mahidol University, Bangkok, Thailand; 3 Department of Molecular Tropical Medicine and Genetics, Faculty of Tropical Medicine, Mahidol University, Bangkok, Thailand; 4 Ministry of Public Health, Islamic Republic of Afghanistan, Kabul, Afghanistan; 5 Centre for Clinical Vaccinology and Tropical Medicine, Churchill Hospital, University of Oxford, Oxford, UK; 6 Center for Emerging and Neglected Infectious Diseases, Mahidol University, Bangkok, Thailand

**Keywords:** *Plasmodium falciparum*, Malaria, Artemisinin combination therapy, Sulphadoxine-pyrimethamine, Dihydrofolate reductase, Dihydropteroate synthase

## Abstract

**Background:**

Artesunate plus sulphadoxine-pyrimethamine (AS+SP) is now first-line treatment for *Plasmodium falciparum* infection in several south Asian countries, including Afghanistan. Molecular studies provide a sensitive means to investigate the current state of drug susceptibility to the SP component, and can also provide information on the likely efficacy of other potential forms of artemisinin-combination therapy.

**Methods:**

During the years 2007 to 2010, 120 blood spots from patients with *P. falciparum* malaria were obtained in four provinces of Afghanistan. PCR-based methods were used to detect drug-resistance mutations in *dhfr*, *dhps*, *pfcrt* and *pfmdr1*, as well as to determine copy number of *pfmdr1*.

**Results:**

The majority (95.5%) of infections had a double mutation in the *dhfr* gene (C59R, S108N); no mutations at *dhfr* positions 16, 51 or 164 were seen. Most isolates were wild type across the *dhps* gene, but five isolates from the provinces of Kunar and Nangarhar in eastern Afghanistan had the triple mutation A437G / K540E / A581G; all five cases were successfully treated with three receiving AS+SP and two receiving dihydroartemisinin-piperaquine. All isolates showed the *pfcrt* SVNMT chloroquine resistance haplotype. Five of 79 isolates had the *pfmdr1* N86Y mutation, while 52 had *pfmdr1* Y184F; positions 1034, 1042 and 1246 were wild type in all isolates. The *pfmdr1* gene was not amplified in any sample.

**Conclusions:**

This study indicates that shortly after the adoption of AS+SP as first-line treatment in Afghanistan, most parasites had a double mutation haplotype in *dhfr*, and a small number of isolates from eastern Afghanistan harboured a triple mutation haplotype in *dhps*. The impact of these mutations on the efficacy of AS+SP remains to be assessed in significant numbers of patients, but these results are clearly concerning since they suggest a higher degree of SP resistance than previously detected. Further focused molecular and clinical studies in this region are urgently required.

## Background

Malaria remains a significant health concern in Afghanistan with ongoing transmission of both *Plasmodium falciparum* and *Plasmodium vivax* in many northern and eastern provinces [1-3]. Resistance of *P. falciparum* to chloroquine in Afghanistan was first apparent in the late 1980s [4], and by 2002 the clinical efficacy of chloroquine and amodiaquine in eastern Afghanistan was extremely low [5]; neither did the addition of artesunate to amodiaquine provide sufficient efficacy, being associated with an adequate clinical and parasitological response rate of only 72% [5]. During this period the efficacy of sulphadoxine-pyrimethamine (SP) was relatively preserved, with cure rates of 77-92% reported in eastern [5] and northern Afghanistan [6] as well as adjacent areas of Pakistan [7] (only the last study was able to correct for re-infection by PCR). Comparable success rates with SP were also noted in south-east Iran, although here there was also evidence of clinical resistance in a small number of parasites, associated with target mutations in dihydrofolate reductase (*dhfr*) and dihydropteroate synthase (*dhps*) [8]. The relative preservation of SP efficacy, along with increasing evidence for long-term benefits of artemisinin combination therapy (ACT), indicated that artesunate combined with sulphadoxine-pyrimethamine (AS+SP) would prove an effective regimen across the region. AS+SP became first-line treatment for *P. falciparum* infection in several south Asian countries, becoming first-line treatment in Afghanistan in 2004, followed by Iran (2006) and India and Pakistan (2007).

Molecular studies probably provide the earliest warning signs of resistance to the SP component of AS+SP [9,10]. For operational reasons, until recently there have been no molecular data on *P. falciparum* SP resistance markers in isolates obtained within the borders of Afghanistan. Indirect indicators of the likely situation in Afghanistan have been obtained through studies in areas of Pakistan with high influx rates of refugees from Afghanistan. Work in this context, undertaken in 2002–3, indicated high efficacy for AS+SP with no evidence of worsening resistance in terms of molecular markers for SP [7].

Mutations in the *P. falciparum* chloroquine resistance transporter gene (*pfcrt*) and multidrug resistance gene 1 (*pfmdr1*), as well as amplification of *pfmdr1*, are associated with altered responses to a variety of antimalarials, including chloroquine as well as the ACT partner drugs amodiaquine, mefloquine and lumefantrine. This work provides an analysis of molecular markers of drug resistance to SP and other anti-malarials in isolates from several sites across Afghanistan.

## Methods

Blood samples were obtained from patients presenting between July 2007 and February 2010 at malaria control centres in the provinces (towns) of Nangarhar (Jalalabad), Kunar (Asadabad), Faryab (Maimaneh) and Takhar (Taloqan). The patients were adults and children over six months old presenting with symptomatic, uncomplicated, microscopically confirmed mono-infection with asexual stages of *P. falciparum*. Blood samples were collected on filter paper (Whatman 3MM) at enrolment. Each filter paper was dried and individually stored in a plastic bag containing silica gel. All filter papers were subsequently transported to the Faculty of Tropical Medicine, Mahidol University, Bangkok, Thailand. Parasite DNA from dried blood spots was extracted via the QIAmp DNA Mini kit using the standard protocol and stored at −20°C until use. PCR-RFLP directed at specific mutations in *dhfr*, *dhps*, *pfcrt*, *pfmdr1* as well as assessment of *pfmdr1* copy number was undertaken using previously described techniques [11-14]; amplification was not always successful, reflecting differing sensitivities of each PCR. In addition, samples with multiple mutations in *dhps* (according to RFLP) were sequenced with the primer corresponding to the published sequences R2 and R/ [11] to confirm multiple mutant genotypes. For the purposes of analysis, in five samples where mixed wild-type / mutant alleles were found at a single locus, the allele was classified as mutant.

The study was approved by the Ethics Committee of the Faculty of Tropical Medicine, Mahidol University, Thailand, the Oxford Tropical Research Ethics Committee, Oxford University, UK and the Institutional Review Board of the Afghan Public Health Institute, Ministry of Public Health, Afghanistan. The work described was undertaken within an overall framework of studies described at the clinicaltrials.gov website under NCT00682578.

## Results

**Figure 1:**
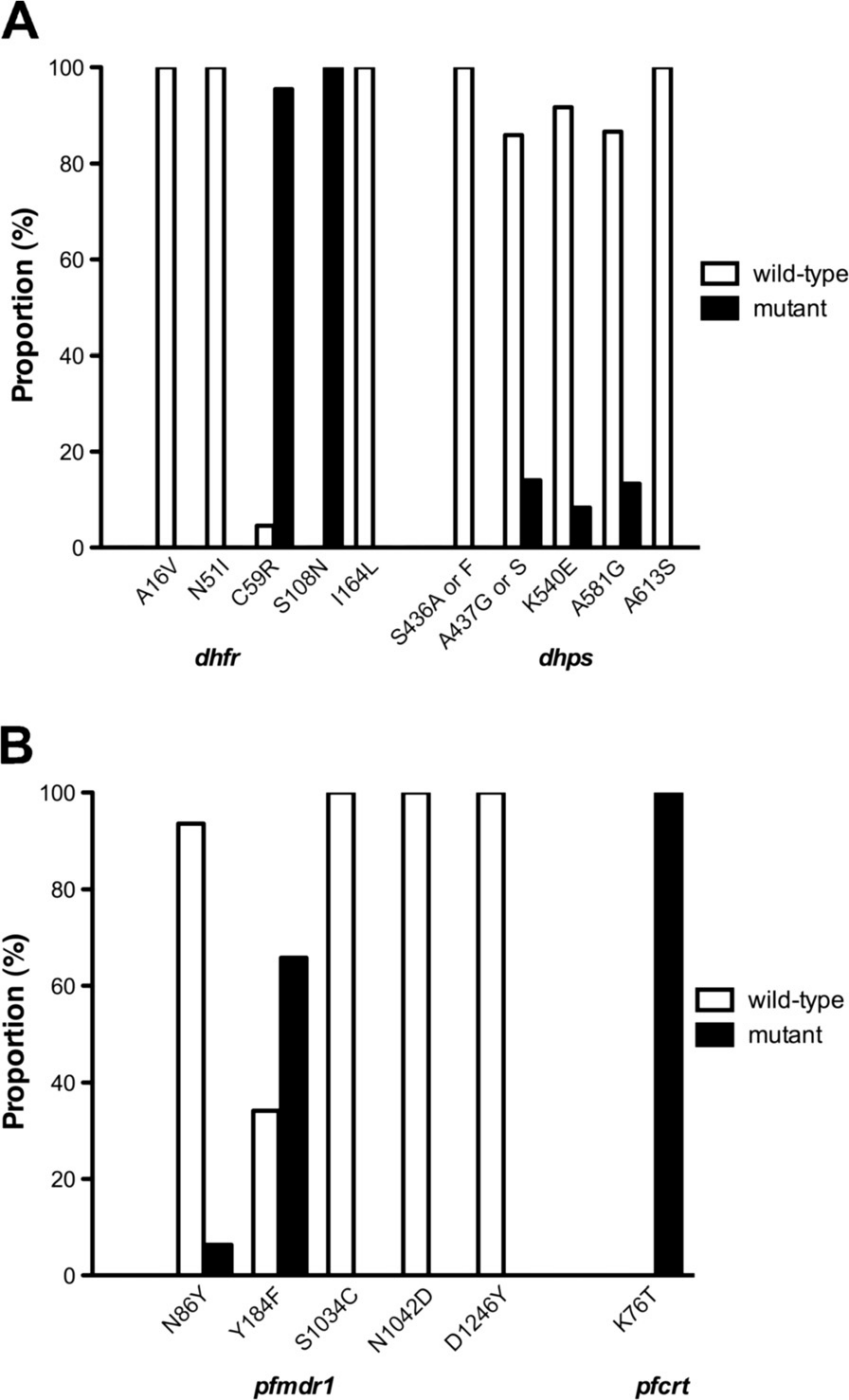
**Prevalence of drug-resistance alleles in isolates of *Plasmodium falciparum *obtained from Afghanistan in the years 2007–2010. A**: SP resistance markers *dhfr* and *dhps*; **B**: relevant *pfmdr1* and *pfcrt* mutations. In all cases samples from the four provinces of Afghanistan obtained in 2007 to 2010 are pooled.

A total of 120 samples were collected, involving 83 patients in Nangarhar, 22 in Kunar, 11 in Takhar and four in Faryab provinces. Individual allele results are shown in Table 1 and Figure 1. More than 90% of genotyped *P. falciparum* infections had a double mutation in the *dhfr* gene (C59R, S108N). No mutations at *dhfr* positions 16, 51 or 164 were seen.

**Table 1:** Numbers of isolates carrying mutations at key positions in four anti-malarial resistance genes

**Gene**	**Mutation examined**	**N**	**Mutated**	**(%)**
DHFR	A16V	44	0	(0)
	N51I	44	0	(0)
	C59R	44	42	(95.5)
	S108N	44	44	(100)
	I164L	44	0	(0)
DHPS	S436A	60	0	(0)
	A437G/S	57	8*	(14)
	K540E	60	5	(8.3)
	A581G	60	8	(13.3)
	A613S	60	0	(0)
PFMDR1	N86Y	79	5	(6)
	Y184F	79	52	(65.8)
	S1034C	85	0	(0)
	N1042D	80	0	(0)
	D1246Y	43	0	(0)
	Copy number**	62	0	(0)
PFCRT	K76T	49	49	(100)

*7 isolates had A437G and1 isolate had A437S; **Mutated = amplified.

In the *dhps* gene, mutations were seen at A437 (7/57 isolates had 437 G and one isolate from Faryab province had 437S), K540E (5/60) and A581G (8/60) (Figure 1A). The *dhps* mutations were clearly linked when examining haplotypes (Table 2), with K540E only seen when both A437G and A581G were present, a similar pattern to that seen in sulphadoxine-resistant parasites from Southeast Asia [15,16]. Furthermore, all five isolates with the triple *dhps* mutant haplotype were obtained in eastern Afghanistan, being derived from Kunar (four isolates) and Nangarhar (one isolate). The patients harbouring these parasites were seen in late 2009 and treated with AS+SP (three cases) and dihydroartemisinin-piperaquine (two cases); all were followed to 42 days without experiencing clinical or parasitological recurrence.

**Table 2:** ***dhfr / dhps *and *pfmdr1 *haplotypes**

**Genes**	**Haplotype**		**Number of isolates**
*dhfr dhps*	ANC**N**I	– – – – –	1
	ANC**N**I	SAKAA	1
	AN**RN**I	SAKAA	21
	AN**RN**I	S**G**KAA	1
	AN**RN**I	S**S**K**G**A	1
	AN**RN**I	S**GEG**A	2
	AN**RN**I	S – KAA	2
	AN**RN**I	S – K**G**A	1
	AN**RN**I	– – – – –	14
	– – – – –	SAKAA	27
	– – – – –	S**G**K**G**A	1
	– – – – –	S**GEG**A	3
*pfmdr1*	NYSND		16
	NYSN –		9
	NYS – –		2
	N**F**SND		23
	N**F**SN –		21
	N**F**S – –		3
	**YF**SND		2
	**YF**SN –		3
	– – SND		2
	– – SN –		4
	– – – – –		5

Haplotypes were determined by the allele at DHFR positions 16, 51, 59, 108 and 164, DHPS positions 436, 437, 540, 581 and 613 and PFMDR1 positions 86, 184, 1034, 1042 and 1246. Mutant alleles are indicated in bold.

All isolates showed the *pfcrt* K76T mutation with no mixed infections (Table 1, Figure 1B). Sequencing in a subset of 28 isolates confirmed the SVNMT *pfcrt* haplotype, consistent with previous studies of *pfcrt* from Afghanistan [17]. Five of 79 isolates had the *pfmdr1* N86Y mutation, and 52/79 had *pfmdr1* Y184F; positions 1034, 1042 and 1246 were wild type in all isolates. The *pfmdr1* gene was not amplified in any sample.

## Discussion

**Figure 2:**
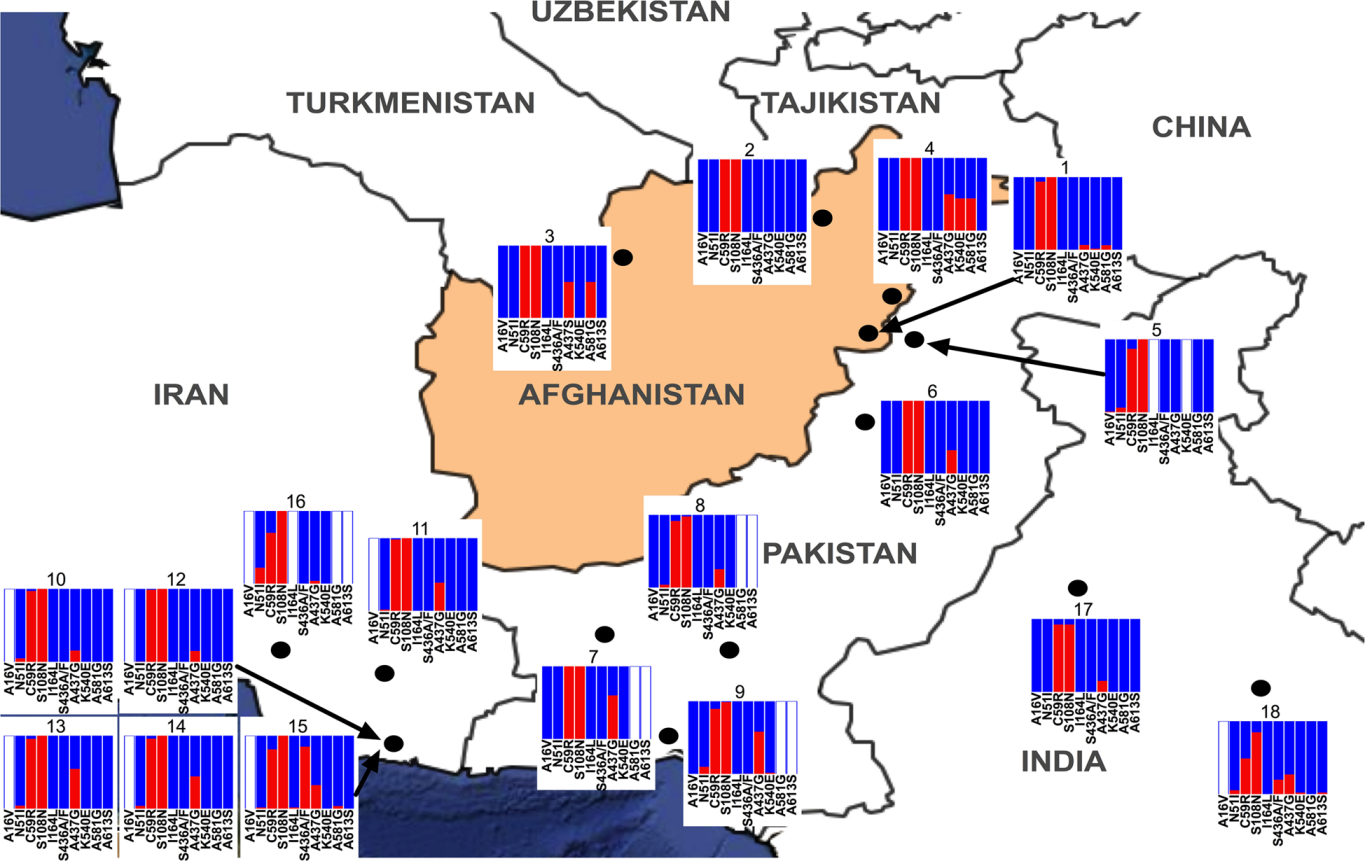
**Regional prevalence of mutant alleles in *dhfr *and *dhps.*** Proportions of isolates with mutations at five positions in *dhfr* (A16, N51, C59, S108, I164) and *dhps* (S436, A437, K540E, A581, A613) are shown for all published molecular studies of SP resistance in south-western Asia (including results described here). Data correspond to the publications described in Table 3, with cross-checking via the Molecular Surveyor website of the Worldwide Anti-malarial Resistance Network (WWARN). Allele frequencies are shown as blue = wild-type, red = mutant, white = not assessed.

A regional overview of published *dhfr* and *dhps* marker data from the last decade, including these new data from Afghanistan along with published data obtained in Iran, Pakistan and northern India (Table 3), reveals a broadly consistent picture (Figure 2). Two mutations in *dhfr* (C59R and S108N) are at or close to fixation throughout the region, but few samples with triple mutations in *dhfr* have been reported, and the critical *dhfr* I164L mutation has remained extremely rare. *dhps* mutations have generally been found only in a minority of samples, and generally as single mutations. However the A437G mutation has been widespread for a considerable time, with samples from southern [18] and north-western [19] Pakistan showing mutation frequencies of 25 to 60%. Previous work in south-east Iran has additionally described relatively high numbers of isolates with S436A/F [8]. From the perspective of combined mutations in *dhfr* and *dhps*, quintuple mutant haplotypes have remained very rare.

**Table 3:** Published molecular surveys of sulphadoxine markers in south-western Asia

**Country**	**State/province**	**Site on map**	**Reference**	**Year of sampling**
Afghanistan	Nangarhar	1	This study	2007–2010
	Takhar	2		
	Faryab	3		
	Kunar	4		
Pakistan	Khyber Pakhtunkhwa	5	[19]	2007
	Khyber Pakhtunkhwa	6	[7]	2000–2003
	Baluchistan, Sindh	7–9	[18]	2005–2007
Iran	Sistan and Baluchistan	10	[20]	2000–2002
		11	[20]	2004
		12	[21]	2003–2004
		13	[22]	2005–2008
		14	[23]	2008–2010
		15	[8]	2003–2005
	Sistan and Baluchistan, Hormuzgan, Kerman	16	[24]	2000–2001
India	Rajasthan	17	[25]	Not stated
	Delhi	18	[26]	1995–2001

This study provides evidence for the presence of parasites with relatively higher levels of SP resistance in eastern Afghanistan, and suggests that molecularly determined SP resistance may be close to compromising the efficacy of first-line treatment for falciparum malaria in Afghanistan. Development of this level of resistance was presumably brought about by the long-term use of SP in the region, since at the time of collection, artemisinin combination therapy (AS+SP) had only just become first-line treatment for falciparum malaria.

There are few data on the clinical response to AS+SP in the setting of double *dhfr* and triple *dhps* mutations, and relatively few patients documented as having this haplotype have entered prospective clinical trials. It is therefore difficult to predict the likely evolution of drug resistance alleles in this area, and much may depend on the field implementation of AS+SP. Unfortunately, there is evidence that inappropriate anti-malarial regimens continue to be prescribed in Afghanistan, including SP alone [27]. This is likely to lead to a further worsening of SP resistance, for example via mutations in *dhfr* (particularly I164L), and an AS+SP combination that increasingly resembles artesunate monotherapy (with the additional risk of emerging artemisinin resistance). In an alternative scenario, consistent use of AS+SP holds the potential to prevent an increase in (or even reduce) the prevalence of SP-resistant parasites, in an analogous manner to that seen for mefloquine [28] and generally predicted for artemisinin combination partners [29]. This type of development was documented in south-east Iran, where the introduction of AS+SP appears to have prevented SP resistance from worsening, and led to a reduction in the proportion of parasites with the 437 G *dhps* mutation [23]. Whatever the relative probabilities of these two outcomes, ongoing studies examining molecular markers as well as clinical efficacy, both in Afghanistan and more widely across the region, are urgently needed.

## Conclusions

Molecular data reveal the presence in eastern Afghanistan of *P. falciparum* parasites with higher levels of SP resistance than previously documented in southern Asia. There is no evidence yet that this is compromising the efficacy of first-line treatment for falciparum malaria in Afghanistan (AS+SP), but ongoing studies examining molecular markers and clinical trials will continue to be important tools in monitoring efficacy, both in Afghanistan and more widely across the region.

## Competing interests

The authors declare that they have no competing interests.

## Authors’ contributions

GRA, SP, AD, ND, NW, CW and MI participated in the conception and design of the study. GRA and FY were responsible for obtaining samples and supervision of study sites. NJ and MI carried out the molecular biology. GRA, CW and MI were responsible for data analysis, data management and drafting of the manuscript. All authors read and approved the final manuscript.
